# The impact of COVID-19 lockdown on the quality of life of Chinese football referees: the chain mediating role of occupational stress and job burnout

**DOI:** 10.1186/s12889-023-16248-9

**Published:** 2023-07-10

**Authors:** Xianliang Wang, Kehao Zong, Yubo Gao, Bochen Li, Shuzhen Wang, Liguo Zhang

**Affiliations:** School of Physical Education, Shandong University, Jinan, 250061 China

**Keywords:** COVID-19 lockdown, Quality of life, Occupational stress, Job burnout, Football referees

## Abstract

**Background:**

COVID-19 lockdown measures have had a great negative impact on the development of sports competition in China, as well as on the quality of life of football referees. This study aims to explore the impact of lockdown measures implemented in response to the COVID-19 pandemic on the quality of life of football referees in China and its mechanism of action.

**Methods:**

The Impact of Event Scale-Revised (IES-R), the Effort–Reward Imbalance Scale (ERI), the Maslach Burnout Inventory General Survey (MBI-GS), and the World Health Organization Quality of Life Brief Version (WHOQOL-BREF). The scale was used from August to September 2022. Using an online questionnaire, 350 questionnaires were sent out and 338 were returned, for a return rate of 96.57%. Invalid questionnaires were excluded, and 307 football referees with referee grades in 29 provinces registered with the CFA were surveyed. SPSS 24.0 and Mplus 8.0 were used for data analysis and structural equation model testing in this study.

**Results:**

The results showed that the COVID-19 lockdown had no significant impact on the quality of life of Chinese football referees. However, the COVID-19 lockdown can affect the quality of life of Chinese football referees through occupational stress or job burnout. Occupational stress and job burnout also play a chain intermediary role between the COVID-19 lockdown and the quality of life of Chinese football referees. In addition, this study further explores the quality of life by dividing it into four dimensions (physical, social, psychological, and environmental). The results show that all four dimensions satisfy the chain mediation model.

**Conclusions:**

Therefore, the quality of life of Chinese football referees can be improved by reducing their occupational stress and job burnout during the COVID-19 lockdown.

## Background

Novel coronavirus pneumonia (COVID-19) is an acute respiratory infection caused by the severe acute respiratory syndrome coronavirus (SARS-CoV-2), first identified in Wuhan, China, in December 2019 [1]. COVID-19 has rapidly spread worldwide since its emergence, presenting a global pandemic and posing a serious threat to human life and health. As of November 7,2022, some 629 million cases had been reported globally, with more than 6.5 million deaths [2]. Therefore, in order to prevent people from contracting COVID-19 and to ensure their health and lives, China has taken measures such as restricting transportation, postponing the resumption of work and school, and suspending or postponing most sports competitions to minimize the flow and gathering of people. Students attended classes at home via the Internet, workers worked at home or temporarily stopped working, and people's habits and quality of life were greatly affected [3–5]. For a profession such as football refereeing, which works mainly on outdoor football pitches, there are even greater difficulties and challenges.

Football referees are an integral part of the game and are needed at all levels and age groups. According to the rules of the game published by FIFA, there must be an on-site referee (FR), two assistant referees (AR), and a fourth official in an official football match. In 2014, there were about 20,000 registered football referees in China [6]. The number of international referees announced by the Chinese Football Association in 2023 is only 32 [7]. Although China has a clear hierarchy of referees and a perfect training management system, due to the late start of the Chinese professional football league and the overall scale of the tournament being on the rise, the Chinese Football Association (CFA) has only implemented a professional referee system for some referees in the Chinese Super League, and most referees are still under the management of the amateur referee system, while, as the Chinese professional football league consists of three tiers of leagues, the high level of football matches are few and the demand for referees is limited, which has led to problems such as a small number of referees, few matches officiated and a lack of competence in China [8]. The salaries of football referees are strongly linked to the number of matches they participate in; however, the new crown pneumonia quarantine policy has led to the postponement or suspension of all sporting events, which has largely reduced their income and increased their financial and survival concerns. As the contagiousness and mortality rate of COVID-19 have declined, some sporting competitions have gradually resumed in China, but all those involved must be isolated for the duration of the competition, leaving football referees not only under pressure from the specificity of their profession but also unable to interact face-to-face and socialize to relieve their fatigue [9]. The accumulation of occupational stress can easily lead to burnout, which can impair the quality of life and physical and mental health of football referees [10–13]. In addition, there are significant challenges in how to continue to develop football in China after the relaxation of the embargo and social distance guidelines, to conduct football matches, and to promote the football referee workforce [14].

The World Health Organization defines the quality of life as the overall satisfaction of individuals in different cultures and value systems with their living conditions concerning their goals, expectations, standards, and concerns, as well as their general sense of personal health [15]. It is a comprehensive measure of well-adjusted physical, psychological, social, and environmental well-being. A large number of studies have shown that during the COVID-19 pandemic, many countries have adopted lockdown and quarantine measures, and people's lifestyles have changed greatly [16–18]. COVID-19 lockdown has reduced people's physical activity and increased sedentary time [19], which may lead to a decrease in the physical fitness and motor skills of referees. Long-term social isolation can lead to feelings of loneliness [20] and even symptoms of insomnia, anxiety, and depression [21]. In addition, isolation makes people unable to communicate with relatives or friends face to face, family support and social support are reduced, and people have nowhere to vent their negative emotions, such as fear and pressure caused by the COVID-19, and cannot get comfort and support from others, which is prone to mental health problems [22]. Therefore, the COVID-19 blockade can have a negative impact on people's quality of life. However, studies in the USA, Poland, and Saudi Arabia found no significant direct effects of COVID-19 on the quality of life of students or adults [23–25]. For soccer referees, they have to travel 10 to 12km to make more than 100 decisions in every soccer match [26]. They must be in good physical and mental condition to make the right decision. On the one hand, the lockdown of COVID-19 has made football referees less physically active and lack organized training and competition, which may lead to a decline in physical fitness and professional skills [27, 28]. On the other hand, because the salary of football referees is related to the number of matches they play, the temporary suspension of work causes them to suffer financial pressure and uncertainty in their career development. This has a great impact on their quality of life [14, 29]. Therefore, hypothesis 1 is formulated: The COVID-19 lockdown harms the quality of life of Chinese football referees.

Stressors refer to environmental demands or threats that individuals find difficult to cope with [30]. According to the theoretical model of stressors proposed by Robbins, potential factors leading to stress include the environment, organization, and individual. When people feel pressure, they will have some physical, psychological, and behavioral symptoms [31]. The COVID-19 is highly infectious, spreads quickly, and the virus antigen is easily mutated. After infection, there will be symptoms such as breathing difficulties, cough, palpitation, muscle soreness, and even life-threatening [32]. COVID-19 lockdown, as a social and environmental stressor, has an important impact on people's physical and mental health and quality of life [33, 34]. Occupational stress refers to the physical and psychological stress caused by the imbalance between objective requirements and self-adaptive ability in the process of work, which is a non-specific abnormal psychological response [35]. The lockdown of COVID-19 has reduced people's physical activity, physical fitness, and sleep quality [36], and they are worried about not being able to recover their previous health status and skill level [37, 38]. For football referees, physical fitness and professional skills are the foundations of their career development. After being quarantined at home for a long time, they will worry about the decline of physical strength and the occupational pressure caused by poor professional skills in the early days of sports competitions [39]. A study of French athletes showed higher anxiety scores when they returned to competition after COVID-19 [40]. Occupational stress is an important factor affecting the quality of life [41]. Long-term, high-intensity occupational stress will cause job burnout, reduce job satisfaction, produce physical and mental health problems [42], and decrease the quality of life [43]. Therefore, hypothesis 2 is formulated: Occupational stress mediates the relationship between COVID-19 lockdown and quality of life.

Burnout is a long-term state of emotional, physical, and mental exhaustion [44, 45], which Maslach et al. believe includes emotional exhaustion, depersonalization, and low personal accomplishment [46]. Job burnout refers to the state of physical and mental fatigue and exhaustion produced by individuals under heavy work pressure, which is defined as a chronic work stress syndrome by the World Health Organization [46]. Previous studies have shown that job burnout is related to sociodemographic and job-related factors, including age, marital status, work experience [47, 48], wage level [49, 50], work stress, social support, etc. [51–53]. According to the job demand-resource model, job demand refers to employees' demands on individuals' physical, psychological, and social abilities in their work. Job resources are factors related to employees' work planning, professional cognition, and organizational development that can promote the realization of employees' work goals. When work demands and work resources cannot match, blindly consuming work resources will lead to job burnout among employees, which will lead to physical and mental health problems, decreased work efficiency, and low job satisfaction [54]. The COVID-19 lockdown has led to the cancellation or postponement of almost all sports matches, and the salary of Chinese football referees is closely related to the number of matches they participate in. Therefore, the COVID-19 lockdown has put football referees under greater economic pressure. In addition, due to the lockdown, football referees have reduced their physical activities, lack normal training and referee performance, and their professional ability is at risk of declining. Therefore, in the early stages of recovery, the lack of professional ability and self-efficacy, as well as the great pressure of refereeing, will lead to job burnout [55, 56], which will affect the physical and mental health and quality of life of the employees [57–59]. Therefore, hypothesis 4 is formulated: Job burnout mediates the relationship between COVID-19 lockdown and quality of life.

A large number of studies have shown that COVID-19 lockdown and isolation measures increase people's fear, stress, anxiety, and depression [60, 61], which have a great impact on their work and lives. For athletes, referees, and other groups who usually have a lot of exercises, the lockdown of COVID-19 makes them have to change their exercise habits and work status and even adjust their expected career plan [62]. The prolonged lockdown and isolation have reduced their physical fitness and professional skill proficiency, which has increased their occupational pressure to some extent. Job burnout is an extreme reaction when individuals cannot cope with work pressure smoothly, and it is a state of exhaustion of emotions, attitudes, and behaviors produced by individuals under long-term pressure [63]. Studies have shown that occupational stress has a direct positive impact on job burnout, and employees who experience job burnout will suffer from decreased work efficiency, physical and mental health problems [64], decreased life satisfaction, and thus reduced quality of life [43, 59]. Therefore, hypothesis 4 is formulated: Occupational stress and job burnout play a chain-mediated role between COVID-19 lockdown and quality of life.

**Fig. 1:**
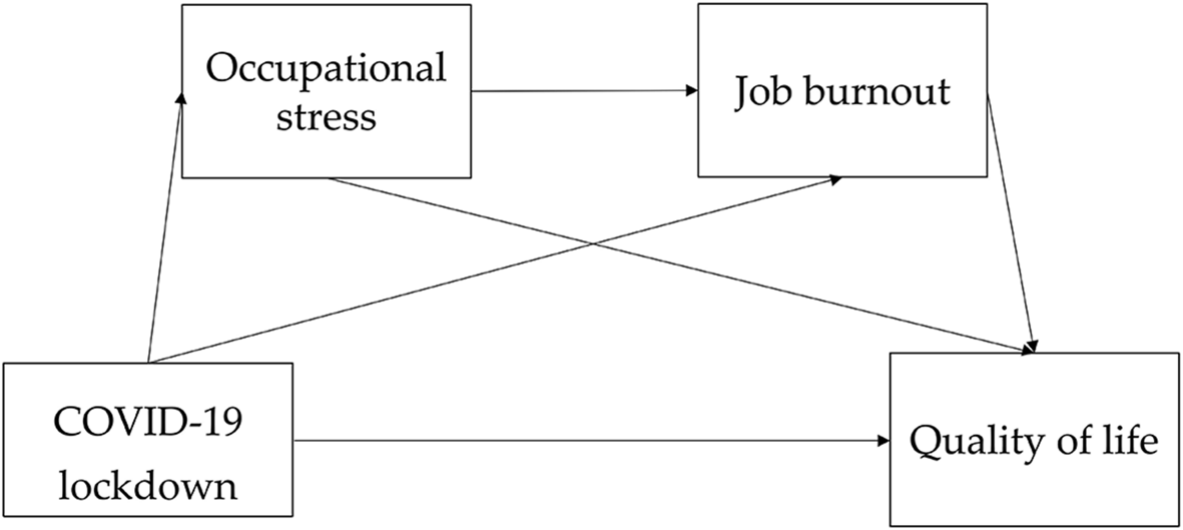
The conceptual framework of this study

In short, the current studies on the impact of the COVID-19 lockdown on quality of life mostly focus on medical staff and students, and most of the studies in the sports industry also focus on athletes, with little research on football referees. To our knowledge, there are no more than 30 studies on the impact of the COVID-19 epidemic on football referees, and most of them focus on physical function, professional skills, and mental health, while there is only one piece of literature on Chinese football referees [39]. Therefore, based on the stressor theory model and the job demand-resource model, this paper explores the impact of the COVID-19 lockdown on the quality of life of Chinese football referees as well as the influencing mechanisms of occupational stress and job burnout. Based on the literature review above, we propose a hypothesis and a hypothesis model (Fig. 1).

## Methods

This is a cross-sectional study using convenience sampling and snowball methods, conducted through an online questionnaire platform (https://www.wjx.cn/) from Au-gust to September 2022. Before completing the questionnaire, participants were in-formed that the survey was anonymous and confidential, and their informed consent was sought. A total of 350 questionnaires were sent out and 338 were recovered, for a recovery rate of 96.57%. The inclusion criteria for the sample were: (1) age ranged from 19 to 45years old and came from 29 provinces in China; (2) referees registered with the CFA at level 3 or above; and (3) taking no less than 10min to complete the questionnaire. During data pre-processing, we removed missing data and invalid samples that took less than 10min to answer the questions and those that did not meet the inclusion criteria, and the final remaining 307 samples were screened. Among them, 254 (82.74%) were male and 53 (17.26%) were female. 108 (35.18%) had been referees for less than three years, 73 (23.78%) for three to five years, 42 (13.68%) for six to eight years, and 84 (27.36%) for more than eight years. There were 40 referees (13.03%) with national referees, 103 (33.55%) with first-class referees, 95 (30.94%) with second-class referees, and 69 (22.48%) with third-class referees. Referees have a high level of skill and have many years of experience refereeing matches in the Chinese Football League. This is a nationally representative sample of data. The survey was approved by the Ethics Committee of the School of Basic Medicine of Shandong University (No. ECSBMSSDU2022-1–086).

### Research instruments

In addition to demographic data such as gender, age, education, and referee performance, the study added four dimensions of quality of life to the hypothesis model: physical domain, psychological domain, social relations domain, and environmental domain.

### Impact of Events Scale-Revised (IES-R)

The Chinese version of the Impact of Event Scale-Revised (IES-R) was used to measure the reactions of Chinese football referees after the COVID-19 lockdown. The scale was developed by Weiss and Marmar [65] and consists of 22 entries with respect to intrusion, avoidance, and hyperarousal. It is rated on a five-point scale from 0 to 4, with 0 being no impact and 4 being severe impact. The main statistical indicator is the total score, which ranges from 0 to 88. According to the instrument, the level of impact can be classified as follows: 0–8 is non-existent, 9–25 is mild, 26–43 is moderate, and > 44 is severe psychological impact [66]. The IES-R scale has been shown to have good reliability and validity in Chinese populations [67]. In this study, Cronbach’s alpha was 0.946.

### Effort–Reward Imbalance Scale (ERI)

The Chinese version of the Effort-Reward Imbalance Scale (ERI) was used to measure the job stress of Chinese football referees. ERI was developed by Siegrist, and the Chinese version of the questionnaire has good reliability and validity [68]. The questionnaire consists of 23 items, of which 6 measure effort, 11 measure reward, and 6 measure overcommitment. All dimensions were rated on a 5-point likert scale, with higher scores reflecting more effort, reward, and over-commitment. The raw score of the reward was obtained through reverse scoring. The total score for each dimension is divided by the number of items to obtain an average score. The ratio between effort and reward scores is an indicator of ERI. Football referees with a ratio > 1 (i.e., high effort and low reward) are defined as highly stressed [69]. The Cronbach’s coefficient of the scale in this study was 0.670.

### Maslach Burnout Inventory General Survey (MBI-GS)

The Chinese version of the Maslach Burnout Inventory General Survey (MBI-GS) [70], which was used to measure the job burnout status of football referees, The scale consists of three sections: emotional exhaustion, cynicism, and reduced personal accomplishment. The emotional exhaustion component consists of 8 questions, the cynicism component consists of 6 questions, and the low sense of accomplishment component consists of 8 questions, for a total of 22 questions throughout the questionnaire. All dimensions are scored on a 5-point likert scale, with 0 being "never" and 6 being "very often". The higher the score, the higher the level of burnout. The scale has been verified to have good reliability and validity in the Chinese population and reports a three-factor structure and good internal consistency (alpha between 0.85 and 0.89) across the sub-scales in China [71]. In this study, Cronbach’s alpha was 0.856.

### World Health Organization quality of life-BREF scale (WHOQOL- BREF)

The Chinese version of the World Health Organization quality of life-BREF scale (WHOQOL-BREF) is used to measure the quality of life of Chinese football referees. The scale is a cross-cultural self-management scale consisting of 26 questions. Quality of life is assessed in four different areas: physical (seven questions), psychological (six questions), social relationships (three questions), and environment (eight questions). Responses varied according to a five-point likert interval scale. Each WHOQOL-BREF item has a score range of 1–5, while each WHOQOL-BREF dimension has a score range of 4–20. A lower score means a poorer quality of life [72]. In this study, Cronbach’s alpha was 0.941.

### Statistical analysis

We used SPSS 24.0 (IBM Corporation, Armonk, NY, USA) and Mplus 8.0 in this study for data analysis and structural equation model testing. The mean and standard deviation were used for all continuous variables, and quantity and percentage were used for categorical variables. First, SPSS was used for descriptive analysis, analysis of variance, and the Pearson correlation test. Then, the structural equation model test was performed using Mplus, using chi-square (2), the goodness of fit index (GFI), TLI, the adjusted goodness of fit index (AGFI), SRMR, and the approximate root mean square error (RMSEA). Finally, Models 4 and 6 from the 3.3 version of PROCESS Macro were used to conduct mediating effects and chain mediating effects tests, respectively. Using the 5000 bias correction Bootstrap method, the mediating effect was significant if the 95% confidence interval (CI) did not contain 0.

## Results

### Common method deviation test

We used the Harman single-factor test to check for common method bias [73]. The results showed that 18 factors all had characteristic roots greater than 1, and the cumulative variance extracted from the first factor was 22.76%, less than 40%. Therefore, there are no serious common methodological biases in this study and it can be continued.

### Descriptive statistics and correlation analysis

**Fig. 2:**
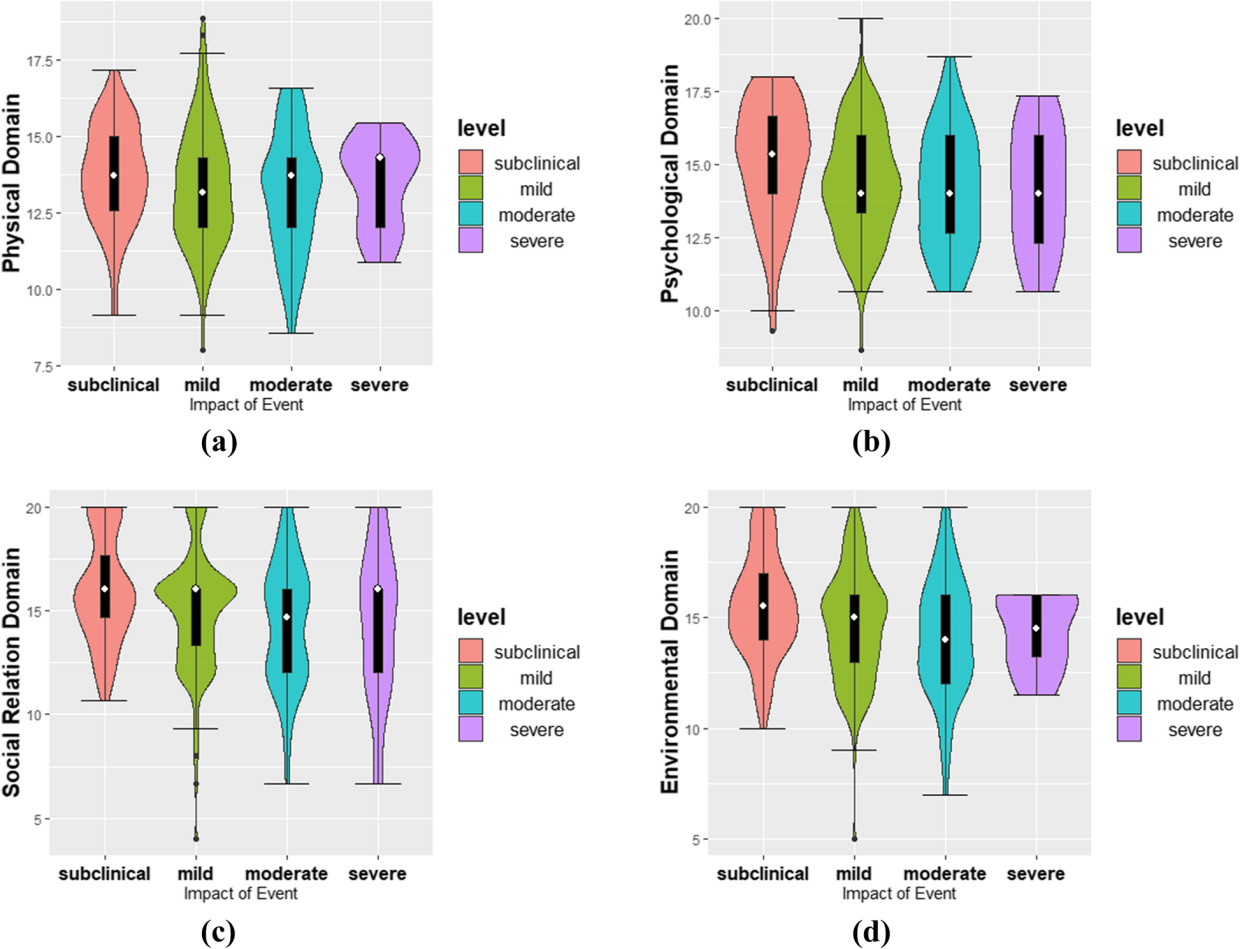
Distribution of the four dimensions of quality of life: **a** Physical domain; **b** Psychological domain; **c** Social relations domain; **d** Environmental domain

Table 1 shows the detailed demographic characteristics of this study. Pearson correlation analysis was conducted for COVID-19 isolation, occupational stress, job burnout, quality of life, and four dimensions of quality of life (physical field, psychological field, social relations field, and environmental field), and the results are shown in Table 2. COVID-19 isolation was positively correlated with occupational stress (*r* = 0.380, *p* < 0.001) and job burnout (*r* = 0.398, *p* < 0.001) and negatively correlated with quality of life (r = -0.301, *p* < 0.001) and its four dimensions. Occupational stress and job burnout were positively correlated with quality of life (*r* = -0.433, *p* < 0.001; *r* = -0.484, *p* < 0.001), and its four dimensions were also significantly negatively correlated. In addition, we also looked at the distribution of the four dimensions of quality of life (see Fig. 2).

**Table 1:** Demographic information

Variables	Item	Number	Percentage (%)
Gender	Male	254	82.74
	Female	53	17.26
Age	19–25	147	47.88
	26–35	83	27.04
	36–45	77	25.08
Level of education (qualification)	High school and below	5	1.63
	College and bachelor's degree	252	82.08
	Master's degree	49	15.96
	Doctoral degree	1	0.33
Level of referee	National level referee	40	13.03
	Grade 1 Referee	103	33.55
	Grade 2 Referee	95	30.94
	Grade 3 referee	69	22.48
Length of experience as a referee	Less than 3 years	108	35.18
	3–5 years	73	23.78
	6–8 years	42	13.68
	Over 8 years	84	27.36
Officiating/assistant referee	Assistant referee	151	49.19
	Officiating referee	156	50.81
Full time/part time	part time	295	96.09
	Full time	12	3.91
Level of physical activity	Light exercise	47	15.31
	Moderate physical activity	85	27.69
	Heavy physical activity	175	57
Total		307	100

**Table 2:** Pearson correlation analysis

		**M**	**SD**	**1**	**2**	**3**	**4**	**5**	**6**	**7**	**8**
1	COVID-19 lockdown	18.153	12.888	1.000	—	—	—				
2	Job burnout	41.404	11.808	0.398^**^	1.000	—	—				
3	Occupational stress	0.216	0.111	0.380^**^	0.421^**^	1.000	—				
4	Quality of life	98.790	14.348	-0.301^**^	-0.484^**^	-0.433^**^	1.000				
5	Physical domain	13.400	1.844	-0.114^**^	-0.266^**^	-0.217^**^	0.709^**^	1.000			
6	Psychological domain	14.508	1.978	-0.181^**^	-0.350^**^	-0.306^**^	0.812^**^	0.628^**^	1.000		
7	Social relations domain	15.053	2.867	-0.236^**^	-0.355^**^	-0.291^**^	0.816^**^	0.613^**^	0.615^**^	1.000	
8	Environmental domain	14.757	2.494	-0.197^**^	-0.392^**^	-0.385^**^	0.906^**^	0.628^**^	0.688^**^	0.695^**^	1.000

^*^p < 0.05, ^**^ *p* < 0.01

We used Mplus 8.0 to test the structural equation model composed of COVID-19 lockdown, occupational stress, job burnout, and quality of life. According to the hypothesis proposed above, the structural equation model was constructed with COVID-19 isolation as the independent variable, quality of life as the dependent variable, occupational stress and job burnout as the mediating variables, and gender and education level as the control variables. As shown in Table 3, the model fitting index was **χ**^**2**^/df = 4; CFI = 0.977; TLI = 0.931; RMSEA = 0.066; SRMR = 0.034. The model fits well. Therefore, hypothesis 1 is valid.

**Table 3:** Model fit index

Index	χ^2^/df	CFI	TLI	RMSEA	SRMR
Result	4	0.977	0.931	0.066	0.034
Ideal value	≤ 5	≥ 0.90	≥ 0.90	≤ 0.08	≤ 0.05

**Fig. 3:**
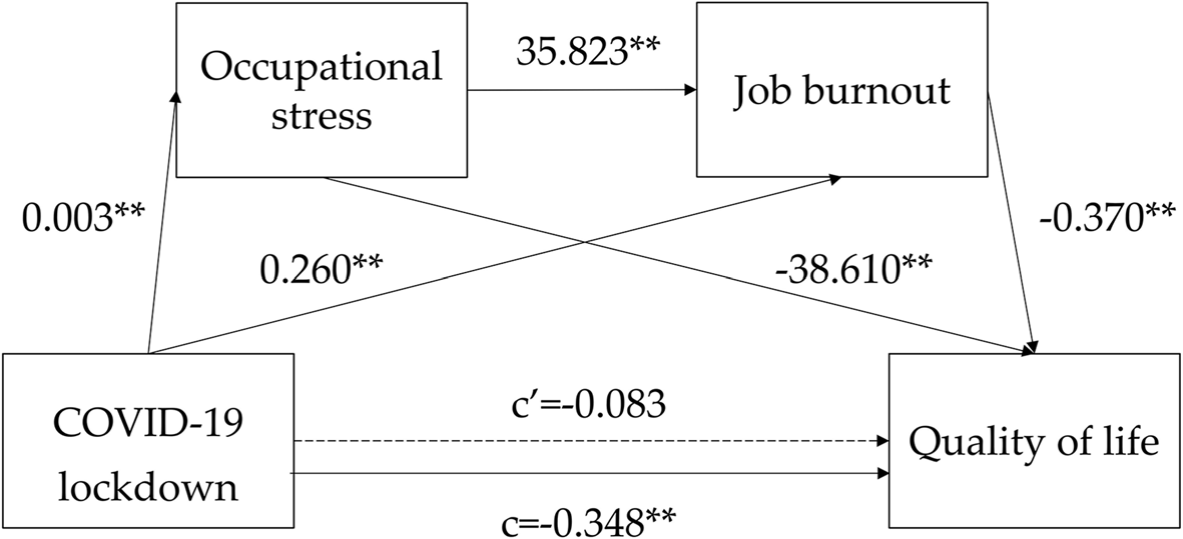
Structural equation modelling

First, we applied models 4 and 6 in PROCESS in SPSS [74] and resampled 5000 times using the deviation-corrected percentile Bootstrap method to calculate the 95% confidence interval for the mediation effect. The results are shown in Table 4. The effect values of three indirect effect paths (COVID-19 lockdown → occupational stress → quality of life, COVID-19 lockdown → job burnout → quality of life, COVID-19 lockdown → occupational stress → job burnout → quality of life) were -0125, -0.096 and -0.043, respectively. Their 95% guiding confidence intervals do not contain 0 ([-0.160,-0.065], [-0.139,-0.042], [-0.063,-0.017]), so the three pathways are significant, that is, occupational stress has an intermediary role between COVID-19 lockdown and quality of life, hypothesis 2 is valid; Job burnout has a unique mediating role between COVID-19 lockdown and quality of life, and hypothesis 3 is valid; Occupational stress and job burnout have a chain mediating effect between COVID-19 lockdown and quality of life, and hypothesis 4 is valid ( Fig. 3).

**Table 4:** Chain mediation effect test

	**Path**	**Effect value**	**Boot SE**	**Boot LLCI**	**Boot ULCI**	**p**
Direct effect	COVID-19 lockdown—Quality of life	-0.083	0.06	-0.2	0.034	0.167
	COVID-19 lockdown—Occupational stress -Quality of Life	-0125	0.024	-0.16	-0.065	0.000
Indirect effect	COVID-19 lockdown—Job burnout -Quality of life	-0.096	0.025	-0.139	-0.042	0.000
	COVID-19 lockdown -Occupational stress- Job burnout -Quality of life	-0.043	0.012	-0.063	-0.017	0.000
Total effect	COVID-19 lockdown—Quality of life	-0.348	0.06	-0.465	-0.23	0.000

**Fig. 4:**
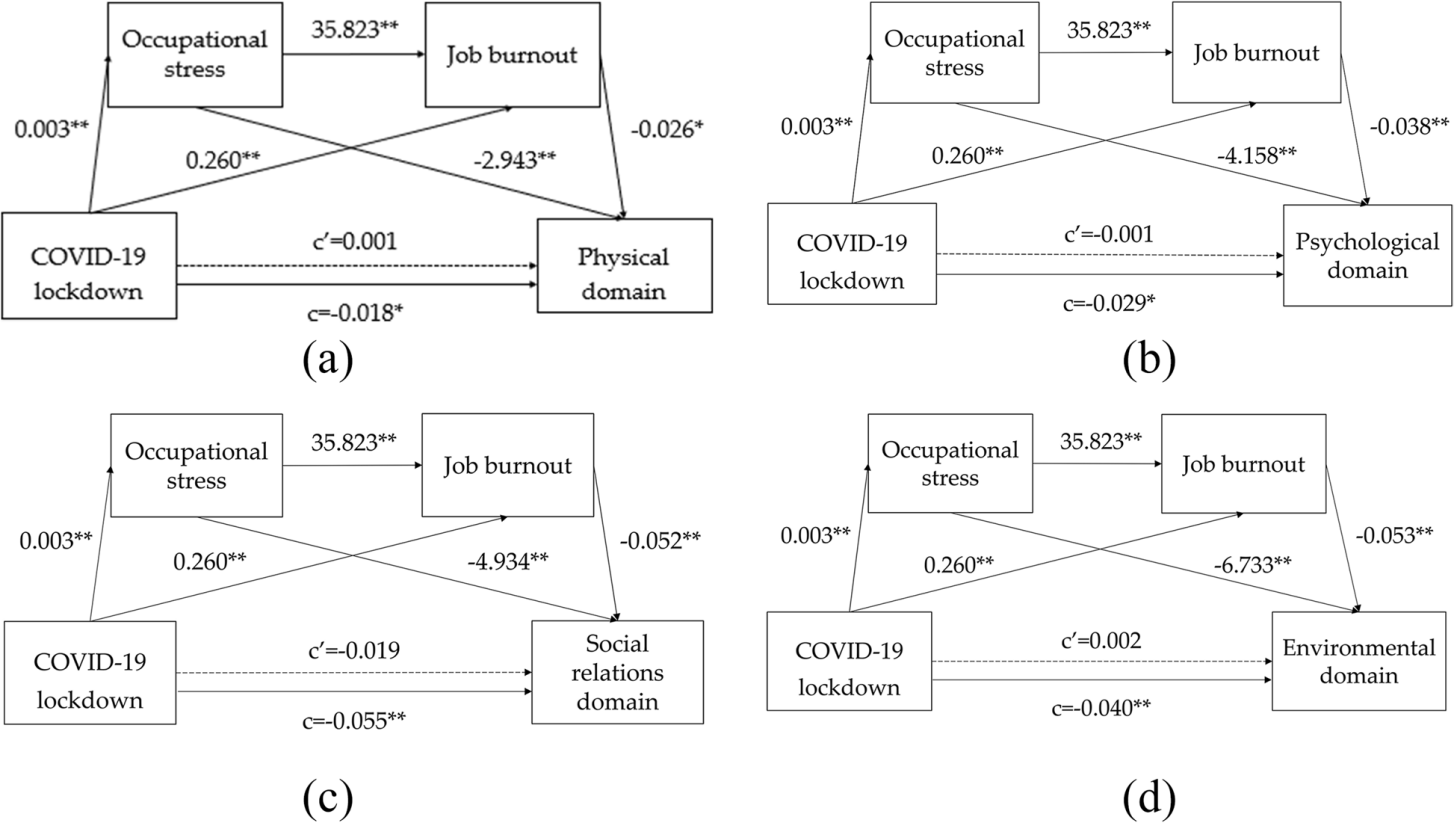
Chain mediation model of the four dimensions of quality of life: **a** Physical domain; **b** Psychological domain; **c** Social relations domain; **d** Environmental domain

To further explore the specific aspects of the COVID-19 lockdown affecting the quality of life through occupational stress and job burnout, this study tested the mediating effect of the four dimensions of the quality of life (physical, psychological, social relations, and environmental) respectively. The analysis results show (Table 5) that the direct effect of COVID-19 lockdown on the four dimensions is not significant, but in the physical field, there are three paths: COVID-19 lockdown → occupational stress → physical field, COVID-19 lockdown → job burnout → physio-logical field, The 95% confidence intervals of COVID-19 lockdown → occupational stress → job burnout → physical field were [-0.114, -0.024], [-0.096, -0.008] and [-0.044,—0.004], excluding 0. Therefore, the COVID-19 lockdown can affect the physical field of Chinese football referees through occupational stress and job burnout. In the psychological field, there are three paths: COVID-19 lockdown → occupational pressure → psychological field, COVID-19 lockdown → job burnout → psychological field, The 95% confidence intervals of COVID-19 lockdown → occupational stress → job burnout → psychological field are [-0.138, -0.037], [-0.113, -0.027] and [-0.053, -0.011], all excluding 0. Therefore, the COVID-19 lockdown can affect the psychological field of Chinese football referees through occupational stress and job burnout. In the field of social relations, there are three paths: COVID-19 lockdown → occupational pressure → social relations field, COVID-19 lockdown → job burnout → social relations field, The 95% confidence intervals of the COVID-19 lockdown → occupational stress → job burnout → social relations field are [-0.122,—0.013], [-0.110, -0.019] and [-0.049, -0.008], excluding 0. Therefore, the COVID-19 lockdown can affect the field of social relations of Chinese football referees through occupational stress and job burnout. In the environmental field, there are three paths: COVID-19 lockdown → occupational pressure → environmental field, COVID-19 lockdown → job burnout → environmental field, The 95% confidence intervals of COVID-19 lockdown → occupational stress → job burnout → environmental field are [-0.164, -0.061], [-0.120, -0.030], and [-0.053, -0.013] respectively, all excluding 0. Therefore, the COVID-19 lockdown can affect the social relations of Chinese football referees through occupational stress and job burnout. To sum up, the four dimensions of quality of life (physical, psychological, social, and environmental) all meet the chain mediation model (Fig. 4).

**Table 5:** Testing the mediating effects of the four dimensions of quality of life (physical domain, psychological domain, social relations domain, environmental domain)

	**Path**	**Effect value**	**Boot SE**	**Boot LLCI**	**Boot ULCI**	**p**
Direct effect	COVID-19 lockdown—physical domain	0.001	0.009	-0.016	0.019	0.879
	COVID-19 lockdown—psychological domain	-0.001	0.009	-0.019	0.016	0.874
	COVID-19 lockdown—social domain	-0.019	0.013	-0.045	0.006	0.143
	COVID-19 lockdown—environmental domain	0.002	0.011	-0.02	0.024	0.877
Indirect effect	COVID-19 lockdown—occupational stress—physical domain	-0.010	0.023	-0.114	-0.024	0.676
	COVID-19 lockdown—job burnout—physical domain	-0.007	0.022	-0.096	-0.008	0.761
	COVID-19 lockdown—occupational stress—job burnout—physical domain	-0.003	0.010	-0.044	-0.004	0.768
	COVID-19 lockdown—occupational stress—Psychological domain	-0.013	0.026	-0.138	-0.037	0.598
	COVID-19 lockdown—job burnout—psychological domain	-0.010	0.022	-0.113	-0.027	0.650
	COVID-19 lockdown—occupational stress—job burnout—psychological domain	-0.004	0.011	-0.053	-0.011	0.680
	COVID-19 lockdown—occupational stress—social domain	-0.016	0.028	-0.122	-0.013	0.566
	COVID-19 lockdown—job burnout—social domain	-0.014	0.023	-0.110	-0.019	0.558
	COVID-19 lockdown—occupational stress—job burnout—social domain	-0.006	0.010	-0.049	-0.008	0.563
	COVID-19 lockdown—occupational stress—environmental domain	-0.022	0.026	-0.164	-0.061	0.400
	COVID-19 lockdown—job burnout—environmental domain	-0.014	0.023	-0.120	-0.030	0.552
	COVID-19 lockdown—occupational stress—job burnout -environmental domain	-0.006	0.010	-0.053	-0.013	0.558
Total effect	COVID-19 lockdown—physical domain	-0.018	0.008	-0.034	-0.002	0.025
	COVID-19 lockdown—psychological domain	-0.029	0.009	-0.046	-0.013	0.001
	COVID-19 lockdown—social domain	-0.055	0.012	-0.079	-0.031	0.000
	COVID-19 lockdown -environmental domain	-0.040	0.011	-0.061	-0.019	0.000

## Discussion

The main purpose of this study is to reveal the impact of the COVID-19 lockdown on the quality of life of Chinese football referees and the mediating role of occupational stress and job burnout. The results showed that the COVID-19 lockdown had no significant effect on the quality of life of Chinese football referees, but the COVID-19 lockdown could indirectly affect the quality of life of Chinese football referees through occupational stress and job burnout. In addition, the four dimensions of quality of life (physical field, psychological field, social relations field, and environmental field) also meet the chain mediation model.

It has been shown that COVID-19 does not have a significant direct effect on people's quality of life. This is consistent with the findings of several previous studies [23–25]. Most studies have found that COVID-19 lockdown can reduce people’s physical activity, increase sedentary time [9, 75], cause mental health problems such as stress, anxiety, and depression [76], and reduce the quality of life. Home isolation is also likely to cause family conflict [77], reduce wages at work, and reduce people’s quality of life [78, 79]. However, several studies in the USA, Poland and Saudi Arabia found no significant direct effect of COVID-19 on quality of life in students or adults [23–25]. The results of the present study corroborate the findings of previous studies. We believe that the reasons for this result are the following: On the one hand, the majority of Chinese football referees consider refereeing their second career. Of the 307 referees included in this study, only 12 were full-time referees. Therefore, although the COVID-19 lockdown has suspended all sporting events and reduced the salaries of football referees, they may still have income from other professions, so they are not under great economic pressure. On the other hand, Chinese football referees have a special working environment and work nature, and they need to bear great physical and psychological pressure when they are working. However, the COVID-19 lockdown allows them to rest at home, which relieves their physical and mental pressure to some extent, so it does not have a significant impact on their quality of life.

The results of this study show that occupational stress plays a mediating role be-tween COVID-19 lockdown and the quality of life of Chinese football referees, that is, COVID-19 lockdown affects their quality of life through occupational stress. This is consistent with the results of previous studies. The COVID-19 lockdown has shut down most businesses and forced employees to rest or work from home, which in large part reduces their work skills. Therefore, in the early days of returning to work, occupational stress occurs due to the inability of physical conditions or professional skills to adapt to occupational requirements [80, 81]. The results of this study reinforce the theoretical model of stressors proposed by Robbins, that is, when people feel the pressure brought by the environment, organization, or individual, they will have some physical, psychological, and behavioral symptoms [31]. Football referees' professional skills decline due to a lack of organized training and refereeing experience when they are isolated at home. They are worried that they cannot adapt to the high-intensity and high-pressure working state, which will cause occupational stress. Occupational stress can directly affect people’s quality of life, leading to negative physical and psychological consequences such as migraines, insomnia, anxiety, and depression [82] and reducing people's quality of life [43, 83].

The results of this study showed that job burnout played a mediating role between the COVID-19 lockdown and the quality of life of Chinese football referees, that is, the COVID-19 lockdown affected their quality of life through job burnout. This is consistent with previous research results [51, 52, 84]. The results of this study strengthen the job demand-resource model [50]. When the job resources cannot match the job demand, employees will have job burnout, which leads to physical and mental health problems, low job satisfaction, and decreased quality of life [85]. COVID-19 has had a great impact and influence on the sports competition industry in China, changing the work environment and status of sports-related staff greatly. Some of the most influential companies have even resorted to layoffs or salary cuts to cope with the crisis. On the one hand, for football referees, the COVID-19 pandemic has changed their work status, making them change from dynamic work to static work at home. The huge work change makes them feel unadaptable and causes job burnout. On the other hand, the COVID-19 lockdown leads to a decline in their physical fitness and professional skills. In the early stages of the resumption of sports competitions, their work ability fails to meet the job requirements, resulting in low personal achievement and job burnout, which further affects their quality of life.

Finally, this study proved the chain-mediating effect of occupational stress and job burnout between the COVID-19 lockdown and the quality of life of Chinese football referees. This is also consistent with previous research results [59, 86]. Job burnout is caused by the long-term accumulation of occupational stress. When employees experience job burnout, they will lose enthusiasm for work, have low work efficiency, and even have the intention of absenteeism and resignation [86]. At the same time, job burnout will also increase people's anxiety and depression symptoms and harm their physical and mental health and quality of life [64, 87, 88]. Affected by COVID-19 isolation, football referees' working ability decreases, which does not match their actual working ability, thus generating occupational stress. Long-term occupational stress leads to job burnout, which further affects their physical and mental health and quality of life.

To the best of our knowledge, this is the first study on the impact of COVID-19 quarantine on the quality of life of football referees in China. This is mainly because, on the one hand, the number of football referees in China is relatively small compared with other professions. According to statistics, only 32 new international level referees were added to the Chinese Football Association in 2023. Therefore, it is not easy to recruit a large number of football referees as research subjects. On the other hand, football referee is a very important and indispensable role in every football match. However, most of the current research mainly focuses on football players and ignores the importance of football referees. Therefore, our study enrich the stressor theory and the job demand-resource model, fills in the relevant research on Chinese football referees, and explores their occupational stress, job burnout and quality of life according to the reality of COVID-19 isolation, so that people pay more attention to the special group of referees. This helps to provide a theoretical basis for government departments and relevant organizations to take corresponding measures to reduce the occupational stress and job burnout of football referees during and after the COVID-19 quarantine, and to ensure that their quality of life is not affected by the COVID-19 quarantine. In addition, we further explore the influencing mechanisms in the four dimensions of quality of life (physical, psychological, social relations, and environmental). Among them, the physical domain included body pain, sleep, daily life and work ability satisfaction. Psychological domains included enjoyment of life, attention, depression, etc. Social relationships include interpersonal relationships, sexual life, and support from friends. Environmental fields include living conditions, medical care, transportation, money, etc. Our results find that the chain mediation model is also valid on these four dimensions. Therefore, alleviating the work stress and/or burnout of football referees can reduce their physical and mental injuries, promote physical and mental health, and improve their social relationship satisfaction, life satisfaction, and well-being. This will play an important positive role in improving the health and well-being of Chinese football referees.

There are also some limitations to this study. First, the study is cross-sectional and cannot explain cause-and-effect relationships between variables. Therefore, we hope that future studies may consider using longitudinal research methods to further explore Secondly, this study used an online questionnaire platform and self-report to collect data. In the future, it is hoped that researchers can conduct research using more objective and multi-angle methods. In addition, our study explored the impact of the COVID-19 lockdown on the quality of life of Chinese football referees from the perspective of occupational stress and job burnout, and there are many unexplored factors. It is hoped that the next research can explore more mediating or moderating variables and enrich its mechanism of action.

## Conclusion

Based on the stressor theory and the demand-resource model, this study takes Chinese football referees as the research objects to explore the impact of COVID-19 lockdown on their quality of life and its mechanisms. The results showed that the COVID-19 lockdown had no significant effect on the quality of life of Chinese football referees, but it could affect the quality of life and the four dimensions of the quality of life (physical field, psychological field, social relations field, and environmental field) through occupational stress and job burnout, respectively. In addition, it can also influence the quality of life and its four dimensions through the chain-mediating effect of occupational stress and job burnout. Therefore, this study provides a theoretical reference for helping Chinese football referees improve their quality of life after the COVID-19 lockdown ends.

## Data Availability

The dataset used and analyzed during the current study are available from the corresponding author on reasonable request.

## References

[1] Merad M, Blish CA, Sallusto F, Iwasaki A (2022). The immunology and immunopathology of COVID-19. Science.

[2] World Health Organization. WHO coronavirus (COVID-19) dashboard. 2023. https://covid19.who.int/. Accessed 14 Feb 2023.

[3] Koc ER, Demir AB, Topaloglu E, Turan OF, Ozkaya G (2022). Effects of quarantine applied during the COVID-19 pandemic on mental health and quality of life in patients with multiple sclerosis and healthy controls. Neurol Sci.

[4] Rogers AM, Lauren BN, Woo Baidal JA, Ozanne EM, Hur C (2021). Persistent effects of the COVID-19 pandemic on diet, exercise, risk for food insecurity, and quality of life: a longitudinal study among U.S. adults. Appetite.

[5] Shoychet G, Lenton-Brym AP, Antony MM (2022). The impact of COVID-19 anxiety on quality of life in Canadian adults: the moderating role of intolerance of uncertainty and health locus of control. Can J Behav Sci.

[6] NetEase. China’s referees are only 10% of Japan’s, both in terms of quantity and quality. 2014. http://sports.163.com/special/chinesereferee/. Accessed 14 Feb 2023.

[7] Zhibo8. CFA international list of referees for 2023: 32 in total, Maning, Fu Ming and Zhang Lei listed. 2023. https://www.toutiao.com/article/7185032586123543075/?source=seo_tt_juhe. Accessed 14 Feb 2023.

[8] Song Q, Zhang C (2014). A research on the development situation and countermeasure Of Chinese football referees. J Anhui Sports Sci.

[9] Shen J, Zhong H (2009). Analysis of the psychological quality cultivation for football referees and their psychological pressure in violation judging. J Chengdu Sport Univ.

[10] Ingersoll RM (2001). Teacher turnover and teacher shortages: an organizational analysis. Am Educ Res J.

[11] Lederer P, Weltle D, Weber A (2001). Illness-related premature unfitness for work among civil servants in Bavaria - an evaluation in the social medical field. Gesundheitswesen.

[12] Vachon M, Papineau M, Dupuis G, Roberge P (2019). Associations between systemic quality of life and burnout among French Canadian workers. Soc Indic Res.

[13] Schonfeld IS, Ruan D (1991). Occupational stress and preemployment measures of depressive symptoms: the case of teachers. J Soc Behav Pers.

[14] Webb T (2021). The future of officiating: analysing the impact of COVID-19 on referees in world football. Soccer & Society.

[15] World Health Organization (1996). WHOQOL-BREF: introduction, administration, scoring and generic version of the assessment: field trial version.

[16] Wang X, Lei SM, Le S, Yang Y, Zhang B, Yao W (2020). Bidirectional influence of the COVID-19 pandemic lockdowns on health behaviors and quality of life among Chinese adults. Int J Environ Res Public Health.

[17] Lesser IA, Nienhuis CP (2020). The impact of COVID-19 on physical activity behavior and well-being of Canadians. Int J Environ Res Public Health.

[18] Qi M, Li P, Moyle W, Weeks B, Jones C (2020). Physical activity, health-related quality of life, and stress among the Chinese adult population during the COVID-19 pandemic. Int J Environ Res Public Health.

[19] Santos AMD, Rossi FE, de Moura HPDN (2021). COVID-19 pandemic impacts physical activity levels and sedentary time but not sleep quality in young badminton athletes. Sport Sci Health.

[20] Bu F, Steptoe A, Fancourt D (2020). Who is lonely in lockdown? Cross-cohort analyses of predictors of loneliness before and during the COVID-19 pandemic. Public Health.

[21] Rossi R, Socci V, Talevi D, Mensi S, Niolu C, Pacitti F (2020). COVID-19 pandemic and lockdown measures impact on mental health among the general population in Italy. Front Psychiatry.

[22] Liu CH, Stevens C, Conrad RC, Hahm HC (2020). Evidence for elevated psychiatric distress, poor sleep, and quality of life concerns during the COVID-19 pandemic among U.S. young adults with suspected and reported psychiatric diagnoses. Psychiatry Res.

[23] Alyami M, de Albuquerque JV, Krägeloh CU, Alyami H, Henning MA (2021). Effects of fear of COVID-19 on mental well-being and quality of life among saudi adults: a path analysis. Saudi Journal of Medicine and Medical Sciences.

[24] Cahuas A, Marenus MW, Kumaravel V, Murray A, Friedman K, Ottensoser H, Chen W (2023). Perceived social support and COVID-19 impact on quality of life in college students: an observational study. Ann Med.

[25] Szemik S, Gajda M, Gładyś A, Kowalska M (2022). The association between COVID-19 pandemic and the quality of life of medical students in Silesian Voivodeship, Poland. Int J Environ Res Public Health.

[26] Krustrup P, Helsen W, Randers MB, Christensen JF, MacDonald C, Rebelo AN (2009). Activity profile and physical demands of football referees and assistant referees in international games. J Sports Sci.

[27] Moreno-Perez V, Martín-Sánchez M, Coso JD, Luis Felipe J, Courel-Ibañez J, Sánchez-Sánchez J (2021). Impact of COVID-19 lockdown on match-activity and physical performance in professional football referees. Biol Sport.

[28] Castillo D, Cámara J, Castagna C, Yanci J (2017). Effects of the off-season period on field and assistant soccer referees `physical performance. J Hum Kinet.

[29] Chen HC, Horne J (2021). The Covid-19 pandemic and asian football confederation match officials. Sport in society.

[30] Lazarus RS (1991). Emotion and adaptation.

[31] Robbins SP, Judge TA (1997). Organizational behavior.

[32] Centers for Disease Control and Prevention. Post-COVID conditions: information for healthcare providers. 2022. https://www.cdc.gov/coronavirus/2019-ncov/hcp/clinical-care/post-covid-conditions.html. Accessed 14 Feb 2023.

[33] Brooks SK, Webster RK, Smith LE, Woodland L, Wessely S, Greenberg N (2020). The psychological impact of quarantine and how to reduce it: rapid review of the evidence. Lancet.

[34] Vitorino LM, Sousa LMM, Trzesniak C, de Sousa Valentim OM, Yoshinari Júnior GH, José HMG (2022). Mental health, quality of life and optimism during the COVID-19 pandemic: a comparison between Brazil and Portugal. Qual Life Res.

[35] Clough BA, March S, Chan RJ, Casey LM, Phillips R, Ireland MJ (2017). Psychosocial interventions for managing occupational stress and burnout among medical doctors: a systematic review. Syst Rev.

[36] Jurecka A, Skucińska P, Gądek A (2021). Impact of the SARS-CoV-2 coronavirus pandemic on physical activity, mental health and quality of life in professional athletes—a systematic review. Int J Environ Res Public Health.

[37] Clemente-Suárez VJ, Fuentes-García JP, de la Vega MR, Martínez Patiño MJ (2020). Modulators of the personal and professional threat perception of olympic athletes in the actual COVID-19 crisis. Front Psychol.

[38] Mon-López D, García-Aliaga A, Ginés Bartolomé A, Muriarte SD (2020). How has COVID-19 modified training and mood in professional and non-professional football players?. Physiol Behav.

[39] Liu Z, Zhao L, Wang S, Gao Y, Zhang L (2022). The association between occupational stress and mental health among Chinese soccer referees in the early stage of reopening soccer matches during the COVID-19 pandemic outbreak: a moderated mediation model. Int J Environ Res Public Health.

[40] Ruffault A, Bernier M, Fournier J, Hauw N (2020). Anxiety and motivation to return to sport during the French COVID-19 lockdown. Front Psychol.

[41] Chegini Z, Asghari Jafarabadi M, Kakemam E (2019). Occupational stress, quality of working life and turnover intention amongst nurses. Nurs Crit Care.

[42] Nochaiwong S, Ruengorn C, Awiphan R, Ruanta Y, Boonchieng W, Nanta S (2020). Mental health circumstances among health care workers and general public under the pandemic situation of COVID-19 (Home-COVID-19). Medicine.

[43] Shen T, Yun N, Sun X (2019). The status quo of professional benefit of surgical nurses and its correlation with professional identity. Ind Health Occup Dis.

[44] Malach-Pines A (2005). The burnout measure, short version. Int J Stress Manag.

[45] Schaufeli W, Enzmann D (1998). The burnout companion to study and practice: a critical analysis.

[46] Maslach C, Schaufeli WB, Leiter MP (2001). Job burnout. Annu Rev Psychol.

[47] Dewa CS, Loong D, Bonato S, Trojanowski L (2017). The relationship between physician burnout and quality of healthcare in terms of safety and acceptability: a systematic review. BMJ Open.

[48] Molero Jurado MDM, Pérez-Fuentes MDC, Gázquez Linares JJG, Simón Márquez MDM, Martos MÁ (2018). Burnout risk and protection factors in certified nursing aides. Int J Environ Res Public Health.

[49] Ramirez-Baena L, Ortega-Campos E, Gomez-Urquiza JL, Cañadas-De la GR, De la Fuente-Solana EI, Cañadas-De la Fuente GA (2019). A multicentre study of burnout prevalence and related psychological variables in medical area hospital nurses. J Clin Med.

[50] Hayes GM, LaLonde-Paul DF, Perret JL, Steele A, McConkey M, Lane WG (2020). Investigation of burnout syndrome and job-related risk factors in veterinary technicians in specialty teaching hospitals: a multicenter cross-sectional study. J Vet Emerg Crit Care.

[51] Ilić IM, Arandjelović MŽ, Jovanović JM, Nešić MM (2017). Relationships of work-related psychosocial risks, stress, individual factors and burnout-Questionnaire survey among emergency physicians and nurses. Med Pr.

[52] Rostamabadi A, Shouroki FK, Jalilian H, Choobineh A, Azmoon H, Shakerian M (2019). The relationship between work-related psychosocial factors and burnout among Iranian nurses: job demand-control-support model. Med Lav.

[53] Khamisa N, Oldenburg B, Peltzer K, Ilic D (2015). Work related stress, burnout, job satisfaction and general health of nurses. Int J Environ Res Public Health.

[54] Demerouti E, Bakker AB, Nachreiner F, Schaufeli WB (2001). The job demands-resources model of burnout. J Appl Psychol.

[55] Kordi R, Chitsaz A, Rostami M, Mostafavi R, Ghadimi M (2013). Incidence, nature, and pattern of injuries to referees in a premier football (soccer) league: a prospective study. Sports Health.

[56] Llorens S, GarcÍa-Renedo M, Salanova M (2005). Burnout as a result of efficacy crisis: a longitudinal study in secondary school teachers. J Work Organ Psychol.

[57] American Psychiatri Association (2013). Diagnostic and statistical manual of mental disorders: DSM-5™.

[58] Cole AH, Leeming DA (2014). Anxiety. Encyclopedia of psychology and religion.

[59] Li X, Jiang T, Sun J, Shi L, Liu J (2021). The relationship between occupational stress, job burnout and quality of life among surgical nurses in Xinjiang. China BMC Nurs.

[60] Alsalhe TA, Aljaloud SO, Chalghaf N, Guelmami N, Alhazza DW, Azaiez F (2020). Moderation effect of physical activity on the relationship between fear of COVID-19 and general distress: a pilot case study in Arabic countries. Front Psychol.

[61] Gouttebarge V, Ahmad I, Mountjoy M, Rice S, Kerkhoffs G (2022). Anxiety and depressive symptoms during the COVID-19 emergency period: a comparative cross-sectional study in professional football. Clin J Sport Med.

[62] Stambulova NB, Schinke RJ, Lavallee D, Wylleman P (2022). The COVID-19 pandemic and olympic/paralympic athletes’ developmental challenges and possibilities in times of a global crisis-transition. Int J Sport Exerc Psychol.

[63] Maslach C, Leiter MP (2016). Understanding the burnout experience: recent research and its implications for psychiatry. World Psychiatry.

[64] Ahola K, Väänänen A, Koskinen A, Kouvonen A, Shirom A (2010). Burnout as a predictor of all-cause mortality among industrial employees: a 10-year prospective register-linkage study. J Psychosom Res.

[65] Weiss DS, Marmar CR, Wilson JP, Keane TM (1997). The impact of event scale–revised. Assessing psychological trauma and PTSD.

[66] Caiuby AVS, Lacerda SS, Quintana MI, Torii TS, Andreoli SB (2012). Cross-cultural adaptation of the Brazilian version of the Impact of Events Scale-Revised (IES-R). Cad Saude Publica.

[67] Le XTT, Dang AK, Toweh J, Nguyen QN, Le HT, Do TTT (2020). Evaluating the psychological impacts related to COVID-19 of Vietnamese people under the first nationwide partial lockdown in Vietnam. Front Psychiatry.

[68] Li J, Yang W, Cheng Y, Siegrist J, Cho SI (2005). Effort–reward imbalance at work and job dissatisfaction in Chinese healthcare workers: a validation study. Int Arch Occup Environ Health.

[69] Siegrist J, Starke D, Chandola T, Godin I, Marmot M, Niedhammer I (2004). The measurement of effort–reward imbalance at work: European comparisons. Soc Sci Med.

[70] Li Y, Zhang K, Zhao G (2005). Confirmatory factor analysis of job burnout. Psychol Explore.

[71] Moreno-Jiménez B, Rodríguez-Carvajal R, Escobar-Redonda E (2001). La evaluación del burnout profesional. Factorialización del MBI-GS. Un análisis preliminar. Ansiedad y Estrés.

[72] dos Santos CM, Hugo FN, Leal AF, Hilgert JB (2013). Comparison of two assessment instruments of quality of life in older adults. Rev Bras Epidemiol.

[73] Fuller CM, Simmering MJ, Atinc G, Atinc Y, Babin BJ (2016). Common methods variance detection in business research. J Bus Res.

[74] Hayes AF (2013). Introduction to mediation, moderation, and conditional process analysis: a regression-based approach.

[75] Maugeri G, Castrogiovanni P, Battaglia G, Pippi R, D’Agata V, Palma A (2020). The impact of physical activity on psychological health during COVID-19 pandemic in Italy. Heliyon.

[76] French MT, Mortensen K, Timming AR (2020). Psychological distress and coronavirus fears during the initial phase of the COVID-19 pandemic in the United States. J Ment Health Policy Econ.

[77] Mehrsafar AH, Gazerani P, Zadeh AM, Sánchez JCJ (2020). Addressing potential impact of COVID-19 pandemic on physical and mental health of elite athletes. Brain Behav Immun.

[78] Epifanio MS, Andrei F, Mancini G, Agostini F, Piombo MA, Spicuzza V (2021). The impact of COVID-19 pandemic and lockdown measures on quality of life among Italian general population. J Clin Med.

[79] Park KH, Kim AR, Yang MA, Lim S-J, Park JH (2021). Impact of the COVID-19 pandemic on the lifestyle, mental health, and quality of life of adults in South Korea. PLoS ONE.

[80] Chen P, Mao L, Nassis GP, Harmer P, Ainsworth BE, Li F (2020). Coronavirus disease (COVID-19): the need to maintain regular physical activity while taking precautions. J Sport Health Sci.

[81] Sandoval-Reyes J, Idrovo-Carlier S, Duque-Oliva EJ (2021). Remote work, work stress, and work–life during pandemic times: a Latin America situation. Int J Environ Res Public Health.

[82] Santos IS, Griep RH, Alves MGM, Goulart AC, Lotufo PA, Barreto SM (2014). Job stress is associated with migraine in current workers: the Brazilian longitudinal study of adult health (ELSA-Brasil). Eur J Pain.

[83] Allan SM, Bealey R, Birch J, Cushing T, Parke S, Sergi G (2020). The prevalence of common and stress-related mental health disorders in healthcare workers based in pandemic-affected hospitals: a rapid systematic review and meta-analysis. Eur J Psychotraumatol.

[84] Yıldırım M, Solmaz F (2022). COVID-19 burnout, COVID-19 stress and resilience: initial psychometric properties of COVID-19 Burnout Scale. Death Stud.

[85] Van Bogaert P, Timmermans O, Weeks SM, van Heusden D, Wouters K, Franck E (2014). Nursing unit teams matter: impact of unit-level nurse practice environment, nurse work characteristics, and burnout on nurse reported job outcomes, and quality of care, and patient adverse events—a cross-sectional survey. Int J Nurs Stud.

[86] Freudenberger HJ (1974). Staff burn-out. J Soc Issues.

[87] Salmela-Aro K, Savolainen H, Holopainen L (2009). Depressive symptoms and school burnout during adolescence: evidence from two cross-lagged longitudinal studies. J Youth Adolesc.

[88] Center C, Davis M, Detre T, Ford DE, Hansbrough W, Hendin H (2003). Confronting depression and suicide in physicians: a consensus statement. JAMA.

